# SHASI-ML: a machine learning-based approach for immunogenicity prediction in *Salmonella* vaccine development

**DOI:** 10.3389/fcimb.2025.1536156

**Published:** 2025-02-11

**Authors:** Ottavia Spiga, Anna Visibelli, Francesco Pettini, Bianca Roncaglia, Annalisa Santucci

**Affiliations:** ^1^ Department of Biotechnology, Chemistry and Pharmacy, University of Siena, Siena, Italy; ^2^ Competence Center Advanced Robotics and enabling digital TEchnologies & Systems 4.0 (ARTES 4.0), Department of Biotechnology, Chemistry and Pharmacy, University of Siena, Siena, Italy; ^3^ SienabioACTIVE-SbA, Department of Biotechnology, Chemistry and Pharmacy, University of Siena, Siena, Italy; ^4^ School of Medicine and Surgery, University of Milano-Bicocca, Monza, Italy

**Keywords:** *Salmonella*, artificial intelligence, machine learning, vaccines, immunogenicity

## Abstract

**Introduction:**

Accurate prediction of immunogenic proteins is crucial for vaccine development and understanding host-pathogen interactions in bacterial diseases, particularly for Salmonella infections which remain a significant global health challenge.

**Methods:**

We developed SHASI-ML, a machine learning-based framework for predicting immunogenic proteins in Salmonella species. The model was trained and validated using a curated dataset of experimentally verified immunogenic and non-immunogenic proteins. Three distinct feature groups were extracted from protein sequences: global properties, sequence-derived features, and structural information. The Extreme Gradient Boosting (XGBoost) algorithm was employed for model development and optimization.

**Results:**

SHASI-ML demonstrated robust performance in identifying bacterial immunogens, achieving 89.3% precision and 91.2% specificity. When applied to the Salmonella enterica serovar Typhimurium proteome, the model identified 292 novel immunogenic protein candidates. Global properties emerged as the most influential feature group in prediction accuracy, followed by structural and sequence information. The model showed superior recall and F1-scores compared to existing computational approaches.

**Discussion:**

These findings establish SHASI-ML as an efficient computational tool for prioritizing immunogenic candidates in Salmonella vaccine development. By streamlining the identification of vaccine candidates early in the development process, this approach significantly reduces experimental burden and associated costs. The methodology can be applied to guide and optimize both research and industrial-scale production of Salmonella vaccines, potentially accelerating the development of more effective immunization strategies.

## 1 Introduction

*Salmonella*, a rod-shaped, Gram-negative bacterium that belongs to the Enterobacteriaceae family, is the most commonly isolated bacterial agent in foodborne infections, both sporadic and epidemic. It occurs in nature with more than 2600 serovars (Gast and Porter, 2020), associated with a broad spectrum of diseases, ranging from mild gastroenteritis to severe systemic infections, making *Salmonella* a significant pathogen of global concern.

*Salmonella* infections can be categorized into typhoidal and non-typhoidal forms. Typhoidal infections, caused by *S. typhi* and *S. paratyphi*, are responsible for typhoid and paratyphoid fevers, which are systemic illnesses with significant global health implications (Marchello et al., 2019; Garrett et al., 2022). Non-typhoidal infections, typically caused by serovars such as *S. typhimurium* and *S. enteritidis*, predominantly manifest as gastroenteric illnesses and remain the most common form of salmonellosis. In addition to these clinical syndromes, certain non-typhoidal serovars are implicated in invasive infections, known as invasive non-typhoidal *Salmonella* infections (iNTS) (Balasubramanian et al., 2019). The global incidence of *Salmonella*-related diseases is alarmingly high, with particularly severe public health impacts in Africa and Asia, where inadequate access to clean water, poor sanitation, and limited healthcare infrastructure significantly exacerbate the burden of disease (Castro-Vargas et al., 2020; Walker et al., 2023).

In Low- and Middle-Income Countries (LMICs), it is estimated that approximately 17.8 million cases of typhoid fever occur annually (Antillón et al., 2017), with Sub-Saharan Africa alone experiencing a burden of over 100 cases per 100,000 people each year and a fatality rate of 1% (Stanaway et al.,; Van Puyvelde et al., 2023). Furthermore, Africa accounts for 26% of the global typhoid-related mortality, equating to 33,490 lives lost annually (Mogasale et al., 2014). Within Nigeria, the toll is particularly severe, with an estimated 364,791 cases of typhoid fever resulting in 4,232 deaths annually; alarmingly, 68% of these fatalities occur among individuals under the age of 15 (Akinyemi et al., 2018). These statistics underscore the devastating impact of *Salmonella* infections, particularly in vulnerable populations such as children and those residing in resource-limited settings.

Salmonellosis, the most common foodborne illness in humans, is primarily transmitted through the consumption of contaminated water or food. Clinical manifestations typically include nausea, vomiting, abdominal pain, and diarrhea, which may range from mild to severe (Wei et al., 2019). Typhoid fever, caused by *S. enterica* serovar Typhi, poses a significant public health challenge in developing countries, where inadequate water supply and sanitation facilitate its transmission (Stanaway et al.,; Stanaway et al., 2019). The growing emergence of multidrug-resistant strains has further compounded the threat of *Salmonella* infections, rendering standard treatments increasingly ineffective. This alarming trend has prompted the inclusion of *Salmonella* on the World Health Organization’s (WHO) antimicrobial resistance (AMR) high-priority pathogen list, underscoring the urgent need for new treatment strategies and interventions (Acheson and Hohmann, 2001; Kariuki et al., 2015; Baliban et al., 2020; WHO Bacterial Priority Pathogens List, 2024: bacterial pathogens of public health importance to guide research, development and strategies to prevent and control antimicrobial resistance, 2024). Vaccines could be a powerful tool against all major *Salmonella* infections, reducing the reliance on antibiotics and helping to combat AMR (MacLennan et al., 2014; Baliban et al., 2020). However, the existing vaccines for typhoid fever offer only moderate protection and are often costly to produce (Rossi et al., 2016; Syed et al., 2020) and, in addition, there are currently no licensed vaccines available for iNTS or paratyphoid fever (Raoufi et al., 2015).

To address these issues, innovative solutions are imperative. Among these, the integration of artificial intelligence (AI) and machine learning (ML) in the medical field is transforming healthcare by providing powerful tools to tackle complex challenges (Visibelli et al., 2023b). These technologies enable the analysis of extensive datasets, uncovering patterns and insights that were previously inaccessible through traditional methods. By doing so, they improve the understanding of disease mechanisms, resistance trends, and population-specific health disparities, while also supporting advancements in diagnostics, treatment, and patient care (Guerranti et al., 2021; Visibelli et al., 2023a). These computational models are enhancing healthcare systems by fostering interdisciplinary collaboration and enabling real-time data sharing, which is particularly beneficial in managing outbreaks and monitoring antimicrobial resistance. Their applications extend to drug discovery (Frusciante et al., 2022), where they streamline the identification of novel therapeutic candidates (Pettini et al., 2021), accelerate clinical trials, and predict drug efficacy and safety profiles. Additionally, AI-powered platforms are being developed to assist in public health interventions by modeling disease spread, improving vaccine distribution strategies, and identifying at-risk populations with greater accuracy. By bridging gaps in traditional healthcare approaches, AI and ML are not only addressing current medical challenges but also paving the way for a more efficient global health ecosystem.

In this context, the aim of the Siena Hub Against *Salmonella* Infections (SHASI) system is to combine vaccine Research & Development (R&D) and industrial expertise to create new approaches toward the development of multivalent vaccines against *Salmonella* diseases. Here, we present SHASI-ML, a machine-learning-based framework designed to predict immunogenic proteins in *Salmonella*. This approach integrates diverse data sources and computational techniques, offering a comprehensive method for analyzing protein immunogenicity. SHASI-ML incorporates structural, sequence-derived, and engineered features to enhance the accuracy and generalizability of predictions, addressing key limitations of existing methods. Beyond prediction, SHASI-ML addresses critical bottlenecks in vaccine development, including cost reduction and safety enhancement. By streamlining the identification of viable vaccine candidates, this study contributes to precision medicine and global health initiatives, paving the way for innovative solutions to combat infectious diseases.

## 2 Method

### 2.1 Dataset creation

To create a dataset of immunogenic and non-immunogenic proteins, we conducted an exhaustive search in PubMed for papers containing data on novel immunogenic proteins tested on humans up until March 2017. This search gathered information on 317 immunogenic proteins from 47 bacterial microorganisms which were collected from the National Center for Biotechnology Information (NCBI) (Sayers et al., 2022) and the Universal Protein Resource KnowledgeBase (UniProtKB) (The UniProt Consortium, 2024). Selection criteria included availability in the database as of March 2017, ensuring the sequences belonged to strains known to infect humans. We included all available strains for which immunogenicity data could be linked to experimentally validated findings. We considered *Salmonella* strains with complete proteome data and evidence of clinical relevance, narrowing the selection to strains with the most comprehensive and high-quality annotations. For proteins with multiple fragments, isoforms, or duplicates, all fragments, and isoforms were included in the dataset to ensure comprehensive representation. Known epitopes were also explicitly included even when their parent proteins were already present. Non-immunogenic proteins were selected from the same bacterial microorganisms using the Basic Local Alignment Search Tool (BLAST) (Altschul et al., 1990). Proteins showing no sequence identity with known immunogenic proteins were identified as non-immunogenic. Additionally, to prevent bias in length distribution, non-immunogenic proteins were filtered to match the length distribution of the immunogenic proteins.

### 2.2 Protein structure and feature prediction

SCRATCH protein structure and structural feature prediction server (Cheng et al., 2005) was primarily used for the prediction of 3- and 8-state secondary structure information. In addition, it was used to calculate the fraction of exposed residues across 20 relative solvent accessibility cutoffs (ranging from ≥0% to ≥95% in 5% intervals). Mono-, di-, and tri-state frequencies of these residues were extracted as well. Moreover, the product of the fraction of exposed residues and the average hydrophobicity of these exposed residues was computed at each relative solvent accessibility cutoff.

### 2.3 Disordered region analysis

To analyze disordered regions in protein sequences, including protein-binding sites, the DISOPRED server (Ward et al., 2004) was employed. The inclusion of disordered region analysis is supported by research showing that intrinsically disordered proteins tend to elicit weak or even non-existent immune responses (Dunker et al., 2002; MacRaild et al., 2016). This can be attributed to the observation that disordered proteins often adopt well-defined conformations when interacting with other proteins or antibodies (Uversky, 2013), resulting in interactions that, while specific, are of relatively low affinity. Based on these findings, additional engineered features were calculated to provide a more in-depth investigation of the disordered regions.

### 2.4 Machine learning model

The Extreme Gradient Boosting (XGBoost) algorithm (Chen and Guestrin, 2016) was employed for model development. XGBoost is a powerful machine learning technique that produces a predictive learner in the form of a set of weak predictive models, allowing the optimization of an arbitrary differentiable cost function. The method employs the gradient descent algorithm to minimize errors in sequential models. This algorithm was chosen due to its strong performance with structured datasets and its ability to prioritize relevant features during training. Hyperparameters were optimized through a grid search process. The final configuration included 800 estimators with a maximum tree depth of 8. The subsampling ratio for columns and training instances was set at 0.7. L1 regularization was set to 1, while the minimum number of samples per leaf and the minimum samples required to split an internal node were set to their default values (1 and 2, respectively). A feature selection step was not necessary as XGBoost already prioritizes important features while filtering out irrelevant ones during training. The dataset was divided into training and validation sets using a stratified split to ensure a balanced representation of immunogenic and non-immunogenic proteins in both sets. Cross-validation was performed to further validate the model’s robustness.

## 3 Results and discussion

**Figure 1:**
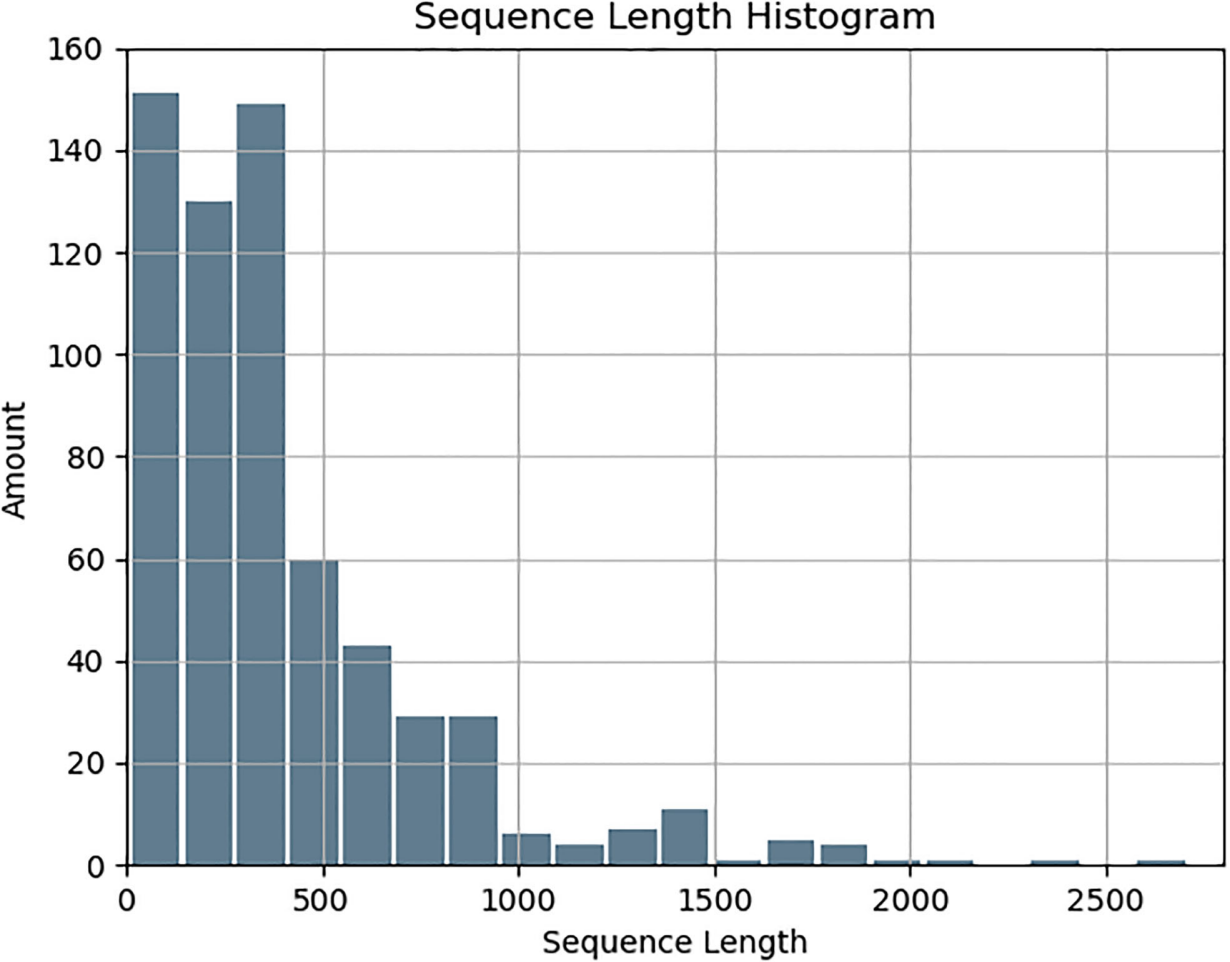
Length distribution of the collected sequences.

Immunogenic proteins were derived from documented human studies, ensuring the inclusion of all available protein fragments and isoforms for a comprehensive dataset. Non-immunogenic proteins were selected using BLAST, maintaining no sequence identity with known immunogenic proteins and ensuring a comparable length distribution. *Salmonella* strains were chosen based on their clinical importance and the availability of high-quality annotations, enhancing the dataset’s relevance to vaccine research. Structural features, including secondary structure and relative solvent accessibility (RSA) predictions, along with engineered metrics such as hydrophobicity and intrinsic disorder, were systematically integrated into the analysis. The collected protein sequences ranged in length from 8 to 2,710 residues, with an average length of approximately 400 amino acids. and a gradual decrease in the sequence length beyond 500 residues, (**Figure 1**).

**Figure 2:**
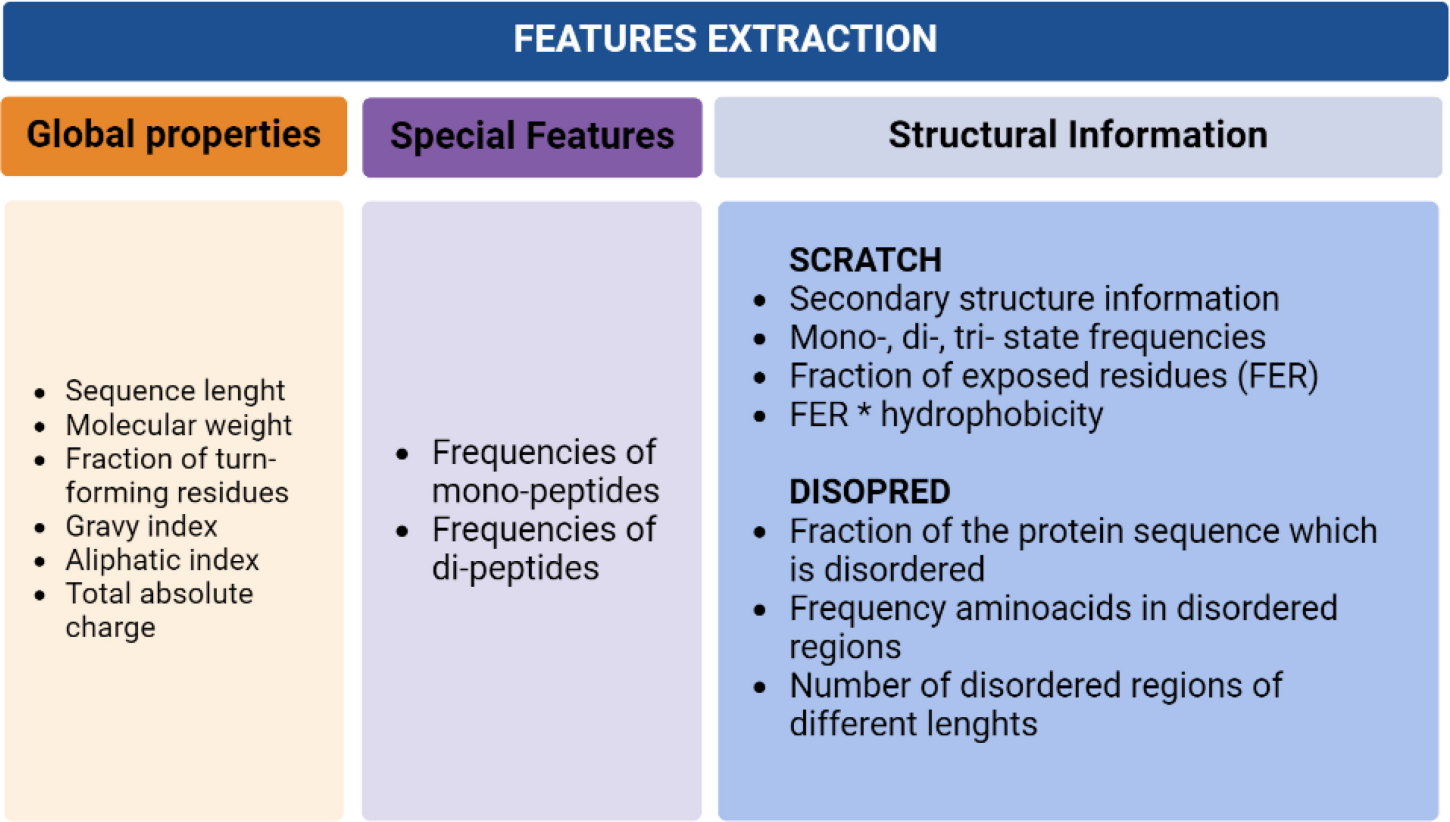
Data pre-processing detailed information.

For data pre-processing, three different groups of features were extracted from each sequence in the dataset. These include global properties of the protein, features derived directly from the protein sequence, and structural information obtained using SCRATCH and DISOPRED. The first set includes molecular weight, sequence length, a fraction of turn-forming residues, the total absolute charge, and the average of hydropathicity and aliphatic indices. The second group of features includes frequencies of mono and di-peptides within the protein sequences. For the third group of features, SCRATCH and DISOPRED were applied, obtaining a total of features able to preserve the relevant information contained in the sequence and reflect the essential properties of the proteins (**Figure 2**).

This information is then used to develop a prediction model of bacterial immunogens based on XGBoost. The network was trained and tested on 20 runs, each using a different dataset split using 80% for the training set and 20% for the test set. The training set included physio-chemical, sequence, and structural properties of 253 immunogenic and 253 non-immunogenic proteins while the test is composed of features of 64 immunogenic and 64 non-immunogenic proteins. Finally, the performance metrics of the model are listed in **Table 1** and include Recall, Specificity, Precision, Accuracy, and F1 score. All these evaluation metrics are based on true positives (TP), true negatives (TN), false positives (FP), and false negatives (FN) outcomes.

**Table 1:** Summary of the performances of SHASI-ML.

Model	TP	TN	FP	FN	Recall	Specificity	Accuracy	Precision	F1 Score
XGBoost	54	55	9	10	0.84	0.86	0.85	0.86	0.84

The metrics include True Positives (TP), True Negatives (TN), False Positives (FP), False Negatives (FN), Recall, Specificity, Accuracy, Precision, and F1 Score, as evaluated using the XGBoost (Extreme Gradient Boosting) model.

Indeed, the SHASI-ML method demonstrated robust performance in identifying bacterial immunogenic proteins. Out of the 128 proteins analyzed, 64 were experimentally validated as immunogenic. The SHASI-ML method correctly identified 54 of these immunogenic proteins, achieving a recall of 0.84. Furthermore, the obtained results highlight that the SHASI-ML model outperforms the best-performing models in (Dimitrov et al., 2020) in terms of Recall, Accuracy, and F1 Score, as shown in **Table 2**.

**Table 2:** Comparison of the performance of the best ML models evaluated in (Dimitrov et al., 2020) and the SHASI-ML.

Model	Recall	Specificity	Accuracy	Precision	F1 Score
RF	0.72	0.82	0.77	0.80	0.76
RSM-1NN	0.72	0.92	0.82	0.91	0.80
XGBOOST	0.84	0.75	0.79	0.77	0.80
SHASI-ML	0.84	0.86	0.85	0.86	0.84

Metrics include Recall, Specificity, Accuracy, Precision, and F1 Score. The models compared are Random Forest (RF), RSM-1NN (Random Subset Method with 1-Nearest Neighbor), XGBoost (Extreme Gradient Boosting), and SHASI-ML.

While the method showed slightly lower precision (0.86) and specificity (0.86) compared to the RSM-1NN method, it compensated for these limitations with higher recall (0.84) and F1-score (0.84), which reflects its ability to minimize false negatives and balance precision with recall. The slight reduction in precision and specificity relative to the RSM-1NN method can be attributed to the broader inclusivity of SHASI-ML, which prioritizes identifying a wider range of true positives. Despite this, the method’s overall superiority is evident in its computational efficiency, ability to handle data inputs, and robust performance across various feature classes. SHASI-ML also reduces the false-positive rate, with only 9 non-immunogenic proteins misclassified as immunogenic.

**Figure 3:**
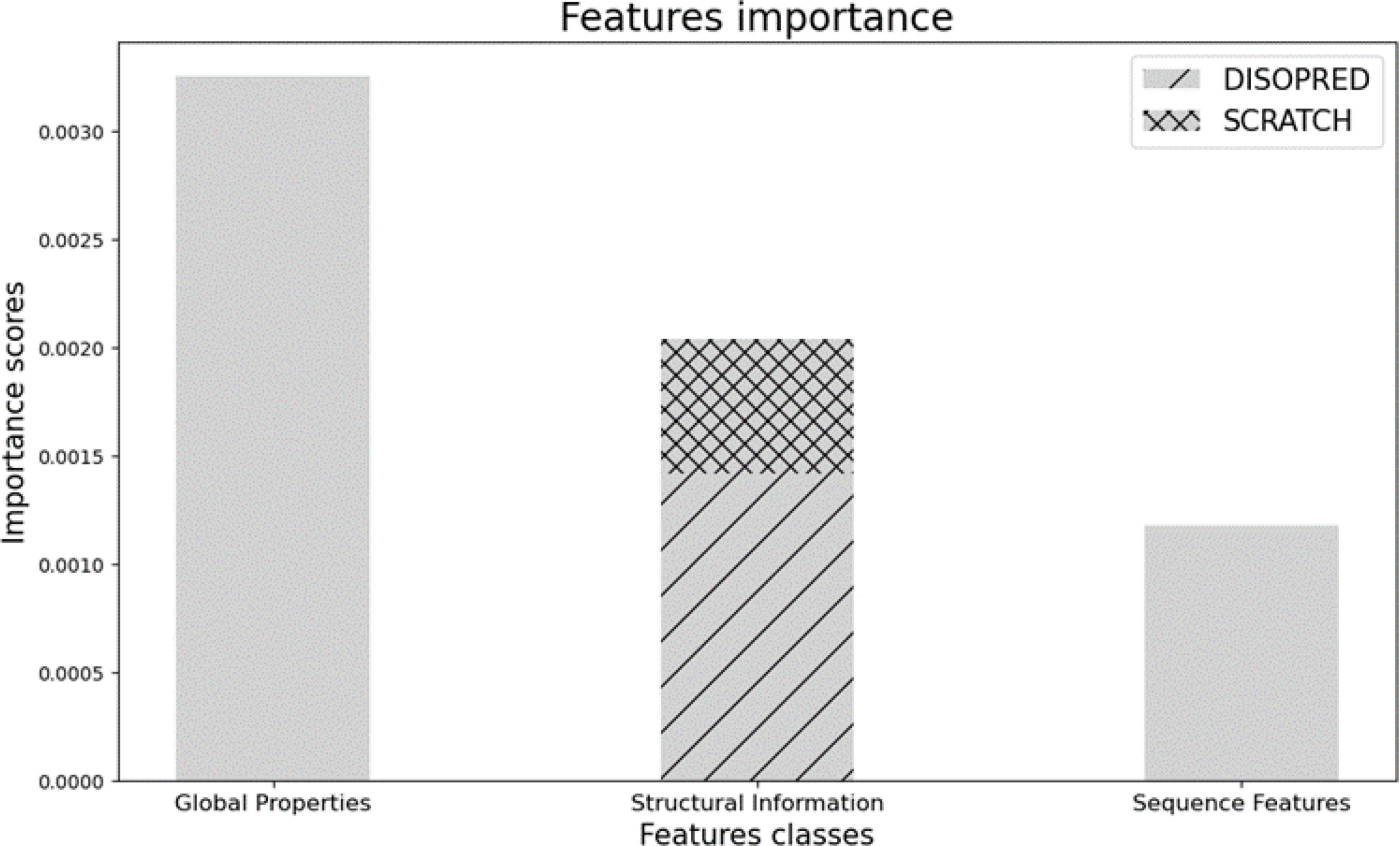
Feature importance scores for predicting bacterial immunogens, grouped by feature classes. The bar chart represents the relative contribution of each feature group (Global Properties, Structural Information, and Sequence Features) toward model performance.

Indeed, our choice of features provides useful information about the physio-chemical, sequence, and structural properties of the protein of interest, which improve prediction performances, showing an outstanding ability to identify bacterial immunogens. We also report the importance of each group of features in the prediction. The feature importance technique assigns a score to the input features based on how useful they are at predicting a target variable. In our case, it indicates how valuable each attribute was in the construction of the boosted decision trees inside the model. The more an attribute is considered to make key decisions, the higher its relative importance. As shown in **Figure 3**, Global properties are the most relevant, followed by Structural and Sequence information, which are less informative for the model.

Finally, an independent dataset of *Salmonella* sequences was created to demonstrate the effectiveness of our model for these protein targets. This dataset was not curated to optimize the model’s performance but rather to evaluate its ability to perform on real-world protein data. We validate the present study by comparing it to the proteome of *Salmonella* enterica serovar Typhimurium (strain LT2/SGSC1412/ATCC 700720) (McClelland et al., 2001) because it shows the best Benchmarking Universal Single-Copy Orthologs (BUSCO) score in the UniProtKB/Swiss-Prot repository. The final dataset contains 1806 protein sequences, from which the three sets of features were extracted. A SHASI-ML prediction was then performed, highlighting new 292 immunogenic proteins suggesting that it can very efficiently select immunogenic proteins from an initial set of candidates reducing time and production costs. Overall, SHASI-ML showed superior performance in immunogenicity prediction, also demonstrating the significance of feature extraction in ML-based prediction.

## 4 Conclusion

The ability of SHASI-ML to predict potential vaccine candidates early in the development process significantly reduces experimental burdens and associated costs, enabling faster and more cost-effective discovery of novel vaccines. This advantage is particularly critical in addressing global health emergencies, where time and resources are often limited. This methodology not only enhances efficiency but also reduces the need for extensive *in vivo* testing, making it a safer option, particularly in regions where immune deficiencies may be latent and undiagnosed among potential vaccine recipients. Additionally, its predictive capabilities allow researchers to focus on the most promising candidates, streamlining the transition from computational analysis to experimental validation.

Looking ahead, these findings will be integrated with the use of modified Outer Membrane Vesicles (mOMVs), a versatile platform designed to further refine and accelerate the R&D process. The synergy between AI-based immunogen identification and mOMV-based platforms holds the potential to significantly advance the development of next-generation vaccines against *Salmonella*. This integrated approach not only increases global health security but also holds promise for advancing vaccine development in low- and middle-income countries, addressing critical global health inequities. The development of a universal vaccine against *Salmonella* serves as a model for applying this methodology to other emerging infectious diseases. Despite these advances, challenges remain. Experimental studies are essential to validate the *in silico* predictions generated by SHASI-ML, and a larger, curated dataset incorporating both positive and negative experimental outcomes will be critical for enhancing prediction accuracy. Future research should also focus on employing advanced ML techniques to further explore the deep immunogenic characteristics of *Salmonella*, paving the way for breakthroughs in vaccine research. By combining innovation, efficiency, and accessibility, SHASI-ML represents a compelling and transformative tool in the fight against infectious diseases, offering a scalable, ethical, and cost-effective pathway to improved global health outcomes.

## Data Availability

Datasets are available on request: The raw data supporting the conclusions of this article will be made available by the authors, without undue reservation.
